# 

**Published:** 1848-07

**Authors:** 


### CONTENTS,

OF THE

### DENTAL REGISTER OF THE WEST.

---

### JULY NO., 1848.

| | | | |
|---|---|---|---|
| Art. | 1. | Valedictory Address, by James Taylor, M. D. D. D. S. | Page 173 |
| " | 2. | Constitution of the Mississippi Valley Association of Dental Sergeons.. | 179 |
| " | 3. | Filling Teeth, by Dr. E. Taylor, | 182 |
| " | 4. | Letter from J. Y. Shirley, Dentist, to St. Louis Editor | 186 |
| " | 5. | The American Society of Dental Surgeons, and the Dental Registor of the West, | 188 |
| " | 6. | Effects of Calomel on the Teeth, by J. Taylor, M. D. D. D. S.—Cin. Ed. | 194 |
| " | 7. | Structure of the Teeth and Extraction thereoff, by Eri Locke, D. D. S... | 199 |
| " | 8. | Physicians Acting the Dentist, by A Berry, D. D. S.—Cin. | 204 |
| " | 9. | Case of Third Dentition in Early Life, by A. Berry, D. D. S.—Cin. | 205 |
| " | 10. | Cast Boxes for Soldering in, | 206 |
| " | 11. | Phrenology, | 207 |
| " | 12. | Whole Sets of Teeth, No. 2, by W. E. Ide, M. D. D. D. S. of Columbns,.. | 108 |
| " | 13. | Dentists in Cincinnati, by Dr. Allen, | 211 |
| " | 14. | Novel Modes of Extracting Teeth, | 212 |
| " | 15. | Filiing Teeth for Children, | 213 |
| " | 16. | Highly Important, | 213 |
| " | 17. | An Infallible Cure for Tooth Ache, | 214 |
| " | 18. | Cutta Percha, | 215 |
| " | 19. | One of the Objects of the Register, | 216 |
| " | 20. | Quack Advertisements, | 216 |
| " | 21. | The Removal of a Foreign Body from the Duct of Wharton,. | 218 |
| " | 22 | Editorial, | 219 |
| " | 23. | Professional Gossip, by Cin. Ed, | 221 |
| " | 24. | Improved Mouth Plates vs. Patents, by Cin. Ed | 223 |
| " | 25. | The Mississippi Valley Asso. of Den. Surg. and the Register, by Cin. Ed. | 225 |
| " | 26. | Waxed Cloth Cones, by Cin. Ed | 225 |
| " | 27. | Hill's Soft Filling, by. Cin. Ed | 226 |
| " | 28. | Ohio College of Dental Surgery, by Cin. Ed, | 226 |
| " | 29. | To Subscribers, &c., &c. | 227 |

